# One-Electron Reduction of Penicillins in Relation to the Oxidative Stress Phenomenon

**DOI:** 10.3390/ijms161226130

**Published:** 2015-12-11

**Authors:** László Szabó, Tünde Tóth, Erzsébet Takács, László Wojnárovits

**Affiliations:** 1 Institute for Energy Security and Environmental Safety, Centre for Energy Research, Hungarian Academy of Sciences, Budapest H-1121, Hungary; takacs.erzsebet@energia.mta.hu (E.T.); wojnarovits.laszlo@energia.mta.hu (L.W.); 2 Department of Organic Chemistry and Technology, Budapest University of Technology and Economics, Budapest H-1111, Hungary; ttoth@mail.bme.hu; 3 Óbuda University, Sándor Rejtő Faculty of Light Industry and Environmental Engineering, Budapest H-1034, Hungary

**Keywords:** antibiotic, penicillin, oxidative stress, reactive oxygen species, reduction, hydrated electron, ketyl radical, pulse radiolysis

## Abstract

Certain bactericidal antibiotics target mitochondrial components and, due to the leakage of electrons from the electron transport chain, one-electron reduction might occur that can lead to intermediates passing the electron to suitable acceptors. This study aimed at investigating the one-electron reduction mechanism of selected penicillin derivatives using pulse radiolysis techniques. Penicillins can accommodate the electron on each of their carbonyl carbon. Ketyl radicals are thus produced, which are reducing agents with possibility to interact with suitable biomolecules. A detailed mechanism of the reduction is reported.

## 1. Introduction

The introduction of antibiotics into medical practice, many decades ago, is regarded as a milestone in the history of medicine saving countless millions of life, thus generating economic welfare. However, we still lack the complete understanding of the action of these long-standing therapeutic agents on bacterial and eukaryotic cells. However, understanding of cell physiology under antibiotic treatment is an important issue for developing new antibiotic therapies that is necessitated by the emerging antibiotic resistance.

Kohanski *et al.* [1] have reported that bactericidal antibiotics (ampicillin, kanamycin, norfloxacin) induce production of reactive oxygen species (ROS), which can contribute to drug mediated cell death in bacteria. The mechanism was shown to imply the rapid depletion of NADH via the tricarboxylic acid cycle inducing an enhanced O_2_^•^^−^ formation via the respiratory chain. O_2_^•^^−^ reduces Fe–S clusters, generating ^•^OH via the Fenton reaction. The theory of a common ROS-mediated killing mechanism of bactericidal drugs was soon challenged by others [2,3], finding that lethality persisted even under anaerobic conditions.

It has also been shown that antibiotics target mitochondrial components in eukaryotic cells (e.g., β-lactam antibiotics interact with the carnitine/acylcarnitine transporter [4]), which phenomenon might be attributed to the putative bacterial origin of mitochondria. Mitochondrial electron transport chain is a main source of ROS in mammalian cells due to the leakage of electrons. Recently, Kalghatgi *et al.* [5] have proved the disruption of mitochondrial function in eukaryotic cells by bactericidal antibiotics resulting in oxidative damage via ROS overproduction.

**Scheme 1:**
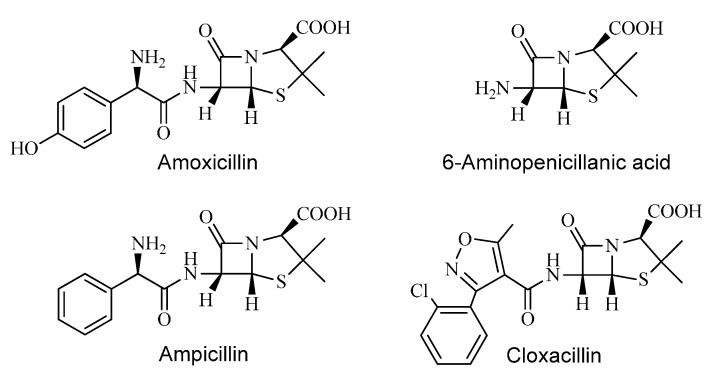
β-lactam derivatives chosen as model compounds.

Redox cycling drugs—quinones, viologens and others—exert their toxic action by abstracting electrons from redox systems and passing them to molecular oxygen generating O_2_^•^^−^ [6]. Our theory suggests a similar mechanism for some bactericidal drugs, which might give a better explanation for the oxidative damage phenomena connected to these molecules. For this purpose, the one-electron reduction mechanism of selected penicillins (amoxicillin, ampicillin, cloxacillin, and their common 6-aminopenicillanic acid sub-structure, Scheme 1) was studied using pulse radiolysis techniques in order to investigate the presence of reactive intermediates that might damage biological systems. The second order rate constants of the reaction of hydrated electrons (e_aq_^−^) with some penicillins have been measured previously by others [7,8] and also in our laboratory [9] addressing different issues. Furthermore, we have studied the one-electron oxidation of amoxicillin previously [10]. The mechanism of the one-electron reduction of penicillin derivatives, however, has not been reported so far.

In this paper we establish a reaction pathway indicating that one-electron reduction of penicillins generates reactive intermediates capable of interacting with biological systems.

## 2. Results and Discussion

### 2.1. Reaction Mechanism of One-Electron Reduction

Hydrated electron attacks electron deficient parts of a molecule, such as a carbonyl carbon. The reactivity of e_aq_^−^ depends on the electrophilicity of the carbon, and therefore, on the adjacent substituents [11]. Penicillins have three carbonyl functions with quite different electronic environment located in the peptide linkage, β-lactam amide bond and carboxyl group (Scheme 1).

The β-lactam carbonyl might be the most capable of accommodating an electron since resonance stabilization cannot decrease the electron deficiency (due to the steric hindrance) as in case of peptidyl carbonyls (e.g., for the simplest carbonyl compound acetone the rate constant of hydrated electron reaction is: *k*e_aq_^−^ = 6.3 × 10^9^ mol^−1^·dm^3^·s^−1^ [11]). In the peptide linkage delocalization implies a lower reaction rate constant (*k*e_aq_^−^ = 1–3 × 10^8^ mol^−1^·dm^3^·s^−1^ for a single peptide group [12]), and the carboxylate group exerts even lower reactivity (*k*e_aq_^−^ = 8.2 × 10^6^ mol^−1^·dm^3^·s^−1^ for zwitterionic glycine + e_aq_^−^ reaction [13]). However, by obtaining ^13^C NMR chemical shifts of the carbonyl carbons of penicillins strikingly close values are achieved, revealing their similar electronic nature (Table 1), which suggests that hydrated electron might target all of these moieties with similar probabilities. Furthermore, in our previous study, e_aq_^−^ efficiently destroyed the β-lactam ring of amoxicillin (84% efficiency), which determines the antibacterial activity, indicating that the hydrated electron attack occurred predominantly at the β-lactam carbonyl [9]. This would be in contradiction with the previous consideration. Here, we will also give explanation for this finding.

**Table 1:** ^13^C NMR characteristics of penicillins.

	^13^C NMR Chemical Shifts of Penicillins		
	Lactam >C=O	Carboxyl –COOH	Peptidyl –C(O)NH–
Amoxicillin	172.93	169.9	169.56
Ampicillin	172.93	170.31	169.42
Cloxacillin	172.18/172.22	169.72	160.41
6-Aminopenicillanic acid	169.86	179.2	–

**Figure 1:**
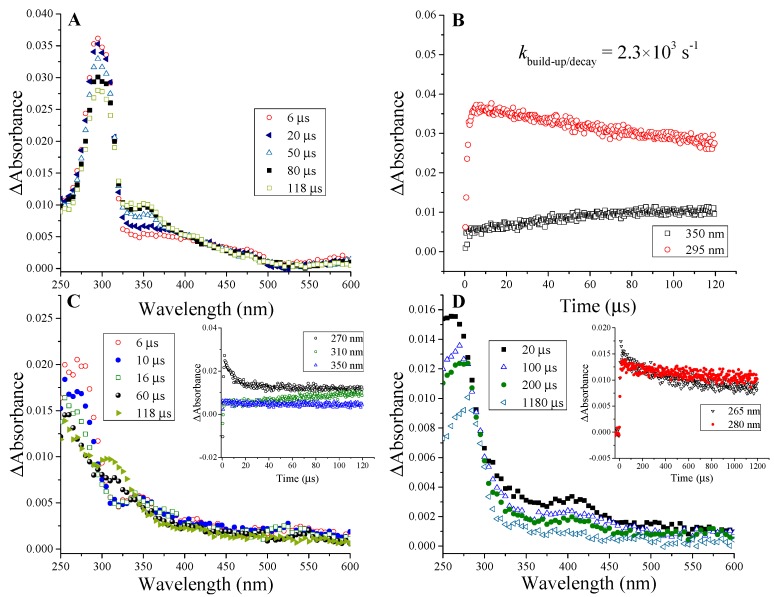
Transient absorption spectra recorded in N_2_-saturated solutions containing 0.5 mol·dm^−3^ *tert*-butanol and 1 × 10^−4^ mol·dm^−3^ ampicillin (**A**); cloxacillin (**C**) with inset showing kinetic traces in a solution as specified for (**C**); and 6-aminopenicillanic acid (**D**) with inset showing kinetic traces in a solution as specified for (**D**); Kinetic trace recorded at 350 and 295 nm in N_2_-saturated solution containing 1 × 10^−4^ mol·dm^−3^ ampicillin and 0.5 mol·dm^−3^ *tert*-butanol (**B**). Dose/pulse was measured to be 20 Gy in each case.

**Figure 2:**
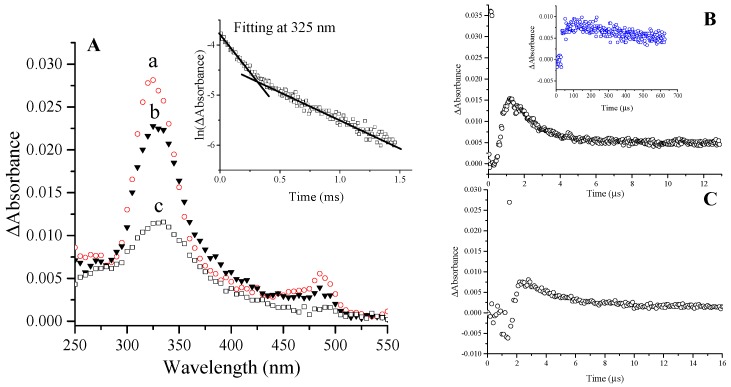
(**A**) Transient absorption spectra of intermediates in N_2_-saturated 1 × 10^−4^ mol·dm^−3^ amoxicillin solution with 0.5 mol·dm^−3^ *tert*-butanol added 10 μs (a); 80 μs (b); and 580 μs (c) after the pulse. Inset: First-order decay fit at 325 nm, rate constants: 2.3 × 10^3^ s^−1^ and 1.1 × 10^3^ s^−1^; (**B**) Kinetic trace recorded at 485 nm (black) with inset showing the trace at 380 nm (blue) in the same solution as specified in (**A**); (**C**) Kinetic trace recorded at 400 nm in N_2_-saturated 2 × 10^−5^ mol·dm^−3^ 6-aminopenicillanic acid solution with 0.5 mol·dm^−3^ *tert*-butanol added. Dose/pulse was measured to be 20 Gy in each case.

The reaction rate constant of the e_aq_^−^ + penicillins reaction was measured following either the build-up of the absorbance at the corresponding λ_max_ of the transient absorption spectra (Figure 1 and Figure 2) or the decay of the e_aq_^−^ at 600 nm. The calculated values were *k*e_aq_^−^ = 5.2 × 10^9^ mol^−1^·dm^3^·s^−1^ and 8.8 × 10^9^ mol^−1^·dm^3^·s^−1^ for amoxicillin and 6-aminopenicillanic acid, respectively, determined previously [9]. These values are different from that determined by Song *et al.* (2008) [8], we attributed this discrepancy to the scavenger technique (see our previous work [9]). Similar values of 5.5 × 10^9^ mol^−1^·dm^3^·s^−1^ are reported herein for both cloxacillin and ampicillin. These close reaction rate constants are quite high values and further confirm a common reaction mechanism of these molecules exhibiting appreciable affinity to accommodate the hydrated electron. The reaction of hydrated electron with water takes place with a reaction rate constant of *k*e_aq_^−^ = 1040 s^−1^ [14]. Penicillins were applied at 1 × 10^−4^ mol·dm^−3^ concentration and even taking the rate constant of 5.2 × 10^9^ mol^−1^·dm^3^·s^−1^ of amoxicillin, 0.19% of the initially available hydrated electron is expected to react with water molecules. Therefore, the effect of this process is neglected during our measurements.

**Scheme 2:**
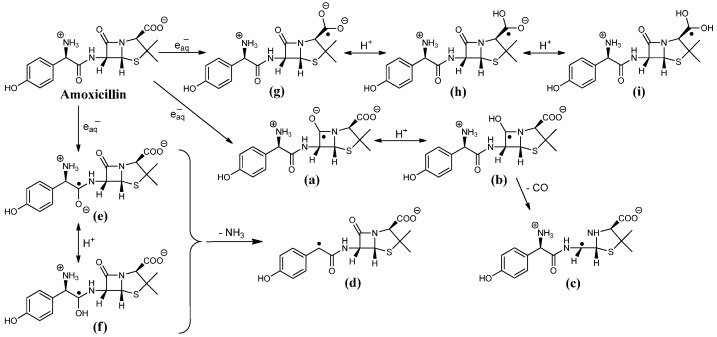
(**a**–**i** radical intermediates) e_aq_^−^ induced reaction pathway of amoxicillin.

The absorption spectra of intermediates produced in the reaction of e_aq_^−^ with penicillins (Figure 1A,C,D and Figure 2A) reveal attack at all the three electrophilic loci and indicate a reaction mechanism depicted in Scheme 2 in case of amoxicillin.

#### 2.1.1. Ketyl Radicals of the β-Lactam Carbonyl

Attachment of the hydrated electron to the β-lactam carbonyl is expected to yield the corresponding ketyl radical anion (Scheme 2**a**).

These radicals (Scheme 2**a**) usually transform immediately (on 10 ns time scale [15]) to α-hydroxyalkyl radicals (Scheme 2**b**) with high p*K*a value of ~11–12 [16]. In the light of our experiments on all the penicillin derivatives (transient absorption spectra of 6-aminopenicillanic acid, ampicillin, cloxacillin and amoxicillin, Figure 1 and Figure 2) the first absorption band (λ_max_ = 270 and 265 nm for cloxacillin and 6-aminopenicillanic acid, respectively) is assigned to the corresponding α-hydroxyalkyl radical (Scheme 2**b**). The band peaking at 295 and 325 nm for ampicillin and amoxicillin, respectively, encompasses two different transient species, one being the corresponding radical mentioned above (Scheme 2**b**). The decay of these absorption bands follows first order kinetics with two consecutive processes (Figure 2A inset). In general, it was always checked that the kinetic trace is pure first order, *i.e.*, it does not show dependence on the radical concentration by varying the dose in the 10–80 Gy range (and this applies throughout the study). In the case of amoxicillin, the faster process is completed within ~80 μs with a rate constant of *k* = 2.7 × 10^3^ s^−1^ and assigned to the disappearance of α-hydroxyalkyl radical (Scheme 2**b**). The initial decay of this band is followed by a parallel increase of the absorbance in the 350–400 nm range (Figure 2A, kinetic trace is shown at 380 nm in Figure 2B inset (blue)), which is in fact the absorption of the forming species generated via this process. The parallel decay and build-up processes can be best observed in case of ampicillin (kinetic trace shown at 350 and 295 nm with *k* = 2.3 × 10^3^ s^−1^, Figure 1B), in case of cloxacillin the forming band is peaking at 310 nm (see Figure 1C inset). The red-shift indicates the migration of the unpaired electron to the adjoining carbon presumably followed by the release of CO eliminating by that the β-lactam pharmacophore (Scheme 2**c**). Such electron migration occurs from ketyl radicals of amino acids and peptides resulting in reductive cleavage of the molecule [17], which eventually yields carbon centered radicals exhibiting also a batochromic shift [18,19]. In the case of 6-aminopenicillanic acid, the decay of the band at 265 nm is much slower and the shift towards longer wavelength after ~1 ms indicates the occurrence of the same process (see Figure 1D inset). The decay of the band assigned to the carbon centered radical (Scheme 2**c**) follows first order kinetics, with *k* = 200, 50 and 30 s^−1^ for ampicillin, 6-aminopenicillanic acid and cloxacillin, respectively, indicating that cloxacillin provides the most stable electronic structure for these species (in case of amoxicillin the decay could not be characterizeddue to the overlap ~380 nm, see Figure 2A).

To observe the spectra of the pure ketyl radicals (Scheme 2**a**) the pH of the solution needs to be adjusted to above pH 11–12. Since penicillins readily hydrolyze under these circumstances [20,21] this was rather avoided and all solutions were prepared at their natural pH.

It is clear that the aromatic ring has an influence on each spectrum. There is a shift to longer wavelength in case of amoxicillin compared to ampicillin and cloxacillin (Figure 1 and Figure 2) that reflects the presence of an electron donating OH group. Furthermore, in cloxacillin (Figure 1C) there is a shift to shorter wavelength compared to ampicillin and amoxicillin (Figure 1A and Figure 2A) in line with the relatively electron-poor aromatic ring. In the latter case we are rather close to the spectra of 6-aminopenicillanic acid (Figure 1D) with absence of the aromatic ring. How the effect of the remote side chain is expressed in the spectral behavior of the ketyl radicals is particularly interesting. We propose that this effect is delivered through the space owing to the possibility of a “coiled” (compact) conformation of the molecules [22,23].

#### 2.1.2. Benzyl Radicals of Ampicillin and Amoxicillin

By analogy to phenylglycine derivatives [24], the remaining absorbance at 325 and 295 nm, 80 and 118 μs after the pulse for amoxicillin and ampicillin, respectively (Figure 1A and Figure 2A), is suggested to belong to the corresponding benzyl radical (Scheme 2**d**). Taking the reported ε = 3.3 × 10^4^ mol^−1^·dm^3^·cm^−1^ for PhCHCOO_2_^−^ a radiation chemical yield of *G* ~0.03 and ~0.04 μmol·J^−1^ can be estimated for the intermediates of this type of amoxicillin and ampicillin, respectively. Therefore, ~11% and 14% of e_aq_^−^ can be predicted to deaminate the molecule. This radical decays following first order kinetics with *k* = 1.1 × 10^3^ and 6 × 10^2^ s^−1^ for amoxicillin and ampicillin, respectively. Addition to the aromatic ring is suggested to be negligible.

#### 2.1.3. Ketyl Radicals of the Peptidyl Carbonyl

We propose that the deamination (in case of amoxicillin and ampicillin) proceeds via the ketyl radical anion (similar to peptides) formed by attachment of the e_aq_^−^ to the peptidyl carbon (Scheme 2**e**). These species are also known to be very weak acids and undergo rapid protonation [13] giving rise to the corresponding α-hydroxyalkyl radicals (Scheme 2**f**). The shape of the spectra with tailing to long-wavelength region (Figure 1A,C and Figure 2A) indicates the presence of these species, which usually exhibit absorption maxima below 240 nm showing monotonous decrease with the wavelength. It follows that deamination via electron transfer from these intermediates (in case of ampicillin and amoxicillin) occurs with less than 100% efficiency (for peptides it is around 80% [25]). Taking an 80% efficiency for the transformation of the α-hydroxyalkyl radical, one can calculate that ~14% and 18% of the initially available hydrated electrons targeted the aromatic ring-containing side chain of amoxicillin and ampicillin, respectively.

#### 2.1.4. Ketyl Radicals of the Carboxylate Group

The carboxylate group on the thiazolidine ring can be regarded as the *C*-terminal residue of the quasi-tripeptide amoxicillin. Indeed, its electronic nature resembles that of amino acids (similar ^13^C NMR chemical shift, Table 1). Although, in amino acids the primary adduct at the carboxyl group is not stable due to the ease of electron migration [17], in the case of benzoic acid the adduct remains for several microseconds and the characteristics of such species was observed [26]. Our findings fairly coincide with the latter study. e_aq_^−^ attack at the carboxylate group generates a radical dianion (Scheme 2**g**). These strongly resonating species possess very high ε value owing to the π → π* transition. The kinetic trace observed at 485 nm (Figure 2B) in case of amoxicillin shows a highly absorbing species right after the pulse, which is immediately depleted. We propose that this diminution is on account of the rapid hydration of the dianion stabilizing that via hydrogen bonds, which event would impair the resonance effect. Owing to the high p*K*a value for the dianion (benzoic acid radical dianion possesses p*K*a = 12 [26]), the subsequent build-up is attributed to the proton abstraction process occurring within 1 μs leading to the intermediate (Scheme 2**h**) (Figure 2B). The radical anion (Scheme 2**h**) exists in equilibrium with the protonated form (Scheme 2**i**) (pH of our solution is 5.2 and p*K*a = 5.3 was reported for benzoic acid radical anion). The protonation causes further decrease in the ε value and takes place within ~4 μs (Figure 2B) with *k*_1_ = 3.3 × 10^5^ s^−1^, which is a slightly slower process than reported in case of the benzoic acid radical anion with *k* = 7.2 × 10^5^ s^−1^ at pH = 5.5 [26]. Decay of the long-living absorption follows first order kinetics with *k*_2_ ≈ 2.9 × 10^3^ s^−1^. According to our findings it appears that the absorption band at shorter wavelengths in case of benzoic acid in the work of Simic and Hoffman [26] can be assigned to the contribution of the aromatic π* orbitals. By taking the reported ε value of the long-wavelength band (ε_435_ = 5200 mol^−1^·dm^3^·cm^−1^) we calculated a radiation chemical yield of *G* = 0.15 μmol·J^−1^ for the radical anion (Scheme 2**h**), which amounts to ~54% of the radiation chemical yield of the hydrated electron. In case of 6-aminopenicillanic acid the same consecutive processes could be observed at 400 nm (Figure 1D, kinetic trace shown in Figure 2C) with *k*_1_ = 3.2 × 10^5^ s^−1^ and *k*_2_ ≈ 2.4 × 10^3^ s^−1^. The blue-shift of the absorption band strongly indicates the effect of the side chain phenyl group (amoxicillin, ampicillin, cloxacillin) and the existence of secondary interactions between the thiazolidine and aromatic moieties. Such interaction is sterically promoted by the “coiled” (compact) conformation of these molecules [22,23]. In the transient spectrum of cloxacillin the peak assigned to these species is even more blue-shifted (peaking at ~350 nm, Figure 1C) in line with the presence of an electron-withdrawing group on the aromatic side chain. In case of ampicillin, species (Scheme 2**i**) absorb around 480 nm.

According to our previous work [9], e_aq_^−^ demolishes the β-lactam system of amoxicillin with 84% efficiency. Since the initial e_aq_^−^ attack occurred with 14% at the side chain, one expects that the first order decay of the long-living absorption (assigned to species (Scheme 2**i**)) implies further electron migration toward the β-lactam nitrogen ultimately yielding β-lactam ring-opening.

#### 2.1.5. General Mechanism of One-Electron Reduction of Penicillins

**Scheme 3:**
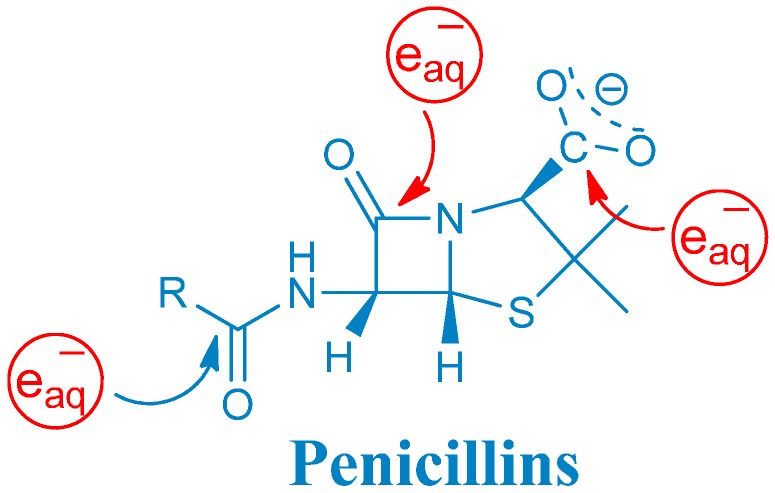
Hydrated electron attack on the penicillins’ skeleton.

It is apparent from the above discussion that a general one-electron reduction mechanism applies to penicillins. A summary table shows the intermediates and some of their features observed in this study (Table 2). The general structure below Table 2 is characteristic of all penicillins (Scheme 3). The hydrated electron attack occurs on this skeleton forming the corresponding ketyl radicals. Taking the example of amoxicillin the hydrated electron prefers these loci (see the structure below) in the following extent: carboxylate carbonyl > β-lactam carbonyl > peptidyl carbonyl.

**Table 2:** Summary of the radical intermediates with a general reaction mechanism below.

	*k*_eaq_	α-Hydroxyalkyl Radicals			Benzyl Radicals
	×10^9^ mol^−1^·dm^3^·s^−1^	β-Lactam Carbonyl	Peptidyl Carbonyl	Carboxylate Carbonyl	
6-Aminopenicillanic acid	8.8 [9]	265 nm; *k* not determined	absorbs < 240 nm	400 nm; *k*_decay_ ≈ 2.4 × 10^3^ s^−1^	no formation
Cloxacillin	5.5	270 nm; *k* not determined		~350 nm	no formation
Ampicillin	5.5	295 nm; *k*_decay_ = 2.3 × 10^3^ s^−1^		~480 nm	295 nm; *k*_decay_ = 1.1 × 10^3^ s^−1^
Amoxicillin	5.2 [9]	325 nm; *k*_decay_ = 2.7 × 10^3^s^−1^		485 nm; *k*_decay_ ≈ 2.9 × 10^3^ s^−1^	325 nm; *k*_decay_ = 6 × 10^2^ s^−1^

## 3. Experimental Section

### 3.1. Materials

Amoxicillin, ampicillin, cloxacillin and 6-aminopenicillanic acid were purchased from Sigma-Aldrich (St. Louis, MO, USA). *Tert*-butanol was provided by VWR International (Radnor, PA, USA).

### 3.2. Methods

Pulse radiolysis experiments were carried out using a Tesla Linac LPR-4 type accelerator (TESLA V.T. Mikroel, Praha, Czech Republic) with kinetic spectrophotometric technique. Single pulses of 4-MeV electrons with duration of 800 ns were used. Samples were irradiated in a 1-cm optical path-length cell using continuous-flow technique. Dose/pulse was measured to be 20 Gy using standard KSCN (Sigma-Aldrich) dosimetry [27], determined before taking each transient spectrum. To obtain kinetic traces an average of at least 20 single measurements were taken to improve the signal/noise ratio. Furthermore, each datum of a spectrum is an average of 5 measurements for the same reasons. The details of the experimental set-up have been described elsewhere [28,29]. The reactions of e_aq_^−^ were studied in N_2_-saturated solution containing 0.5 mol·dm^−3^ *tert*-butanol, which scavenges ^•^OH according to the reaction: ^•^OH + (CH_3_)_3_COH → H_2_O + ^•^CH_2_C(CH_3_)_2_OH, affording a radiation chemical yield of *G*(e_aq_^−^) = 0.28 μmol·J^−1^. The forming *tert*-butyl alcohol radicals are unreactive on the timescale of our measurements and absorb below 300 nm with low molar absorption coefficient (ε_280_ = 30 mol^−1^·dm^3^·cm^−1^, ε_250_ = 200 mol^−1^·dm^3^·cm^−1^ [16]). Solutions were prepared at natural pH of ~5, where aminopenicillins exist in their zwitterionic forms, whereas 6-aminopenicillanic acid and cloxacillin are mainly in monoanionic form (carboxylate).

^13^C NMR spectra (500 MHz) were recorded on a Bruker DRX-500 Avance spectrometer using DMSO-*d*_6_ (TMS).

## 4. Conclusions

Penicillins behave towards e_aq_^−^ somewhat like a tripeptide in line with their structural similarities. The hydrated electron is accommodated on the carbonyl carbons of these molecules. The forming ketyl radicals are reducing agents that might pass the electron to another acceptor (e.g., O_2_). Contrary to peptides, the adduct at the carboxylate group could be stabilized for several μs similarly to benzoic acid. The unique electronic structure of the thiazolidine ring is obviously behind this effect.

It appears to be likely that penicillins accommodate an electron easily and release that to other suitable partner, which might give explanation to the oxidative stress phenomena thoroughly observed for penicillins in the literature (see Introduction). To explore whether the electron can be donated to the O_2_ or other partners the one-electron reduction potential of penicillins needs to be determined.
